# Validation of the Thai version of the obstetric quality of recovery score (obsqor-10-Thai) after elective cesarean delivery

**DOI:** 10.1186/s12871-023-02010-6

**Published:** 2023-03-07

**Authors:** Sasima Dusitkasem, Jinda Jindapitak, Vanlapa Arnuntasupakul, Varinee Lekprasert, Sommart Bumrungphuet, Chakrit Sukying, Rojnarin Komonhirun, Lisa Sangkum

**Affiliations:** 1 270 Department of Anesthesiology, Faculty of Medicine, Ramathibodi Hospital, Mahidol University, Rama VI Road, Phayathai, Ratchatewi, Bangkok, 10400 Thailand; 2 270 Department of Obstetrics and Gynecologics, Faculty of Medicine, Ramathibodi Hospital, Mahidol University, Rama VI Road, Phayathai, Ratchatewi, Bangkok, 10400 Thailand; 3 270 Department of Psychiatry, Faculty of Medicine, Ramathibodi Hospital, Mahidol University, Rama VI Road, Phayathai, Ratchatewi, Bangkok, 10400 Thailand

**Keywords:** Cesarean, Childbirth, Obstetric quality of recovery

## Abstract

**Background:**

The Obstetric Quality of Recovery score (ObsQoR-10) is a questionnaire used to assess recovery after cesarean delivery. However, the original ObsQoR-10 is in English and was mainly validated in the Western population. We therefore evaluated the reliability, validity, and responsiveness of the ObsQoR-10-Thai in patients undergoing elective cesarean delivery.

**Methods:**

The original ObsQoR-10 was translated into Thai, and psychometric validation was performed to evaluate the quality of post-cesarean recovery. The ObsQoR-10-Thai, activities of daily living checklist, and 100-mm visual analog scale of global health (VAS-GH) questionnaires were administered to the study participants before and 24 and 48-h postpartum. Validity, reliability, responsiveness, and feasibility of the ObsQoR-10-Thai were assessed.

**Results:**

We included 110 patients undergoing elective cesarean delivery. The mean ObsQoR-10-Thai score at baseline and 24 and 48-h postpartum was 83.35 ± 11.15, 56.75 ± 11.6, and 70.96 ± 13.65, respectively. The ObsQoR-10-Thai score differed significantly between the two groups divided based on the VAS-GH (≥ 70 vs. < 70): 75.58 ± 13.81 and 52.56 ± 10.61, respectively (*P* < 0.001). The convergent validity between the ObsQoR-10-Thai and VAS-GH was good (*r* = 0.60, *P* < 0.001). The ObsQoR-10-Thai displayed good internal consistency (Cronbach’s alpha = 0.87), split-half reliability (0.92), and test–retest reliability (0.99, 95% CI: 0.98–0.99). The median time to complete the questionnaire was 2 (IQR, 1–6) min.

**Conclusions:**

Our findings indicate that the ObsQoR-10-Thai is valid and has good reliability, with a high degree of responsiveness in terms of assessment of recovery after elective cesarean delivery.

**Trial registration:**

This study was registered on the Thai Clinical Trials Registry, identifier TCTR20210204001, registered on 04/02/2021 (Prospectively registration).

**Supplementary Information:**

The online version contains supplementary material available at 10.1186/s12871-023-02010-6.

## Background

Cesarean delivery is one of the most commonly performed surgeries in the world, with nearly 18.5 million cesarean deliveries performed annually [1]. Recovery after cesarean delivery is a complex process and depends on various patient, surgical, and anesthetic factors, as well as the occurrence of postoperative complications. However, the quality of post-cesarean recovery is usually assessed using limited single-dimensional outcomes (e.g., visual analog scale pain scores and morbidity or mortality rate), which are not comprehensive. Several multidimensional assessments have been used to evaluate recovery after cesarean delivery, such as quality of recovery (QoR)-40 [2], QoR-15 [3], or short-form health survey (SF-36) [4]. However, these scoring systems have not been validated in the obstetric population and do not include obstetrics-specific items (e.g., ability to care for the baby). Multidimensional parameters are therefore crucial for measuring post-cesarean recovery.

The obstetric quality of recovery score (ObsQoR-11) is an 11-item questionnaire that covers four domains of recovery outcomes: physical comfort, emotional state, physical independence and care of the neonate, and pain [5]. However, ObsQoR-11 has been updated to the 10-item ObsQoR-10 by combining severe and moderate pain items, based on patient feedback [6]. The total score can range from 0 to 100, with 0 = worst recovery and 100 = best recovery. The ObsQoR-10 has been tested for validity, reliability, responsiveness, and clinical utility in measuring health status following cesarean delivery and spontaneous or vaginal delivery in multiple healthcare setting [6–13]. A recent extensive scoping review using ‘consensus-based standards for the selection of health measurement instruments’ (COSMIN) criteria to evaluate the quality of patient-reported outcome measures (PROM) instruments that have been utilized to assess postpartum recovery following caesarean section. Overall, the Obstetric Quality of Recovery (ObsQoR) achieved the highest COSMIN standards for any PROM [14]. Moreover, ObsQoR-10 was one of a core outcome set for enhanced recovery after cesarean delivery [15]. Since the ObsQoR-10 is an English language measurement tool that has been validated mainly in Western patients. Therefore, it might not be widely applicable to the Asian population, which has differences in culture, attitude, perception, and lifestyle. In the present study, we translated and validated the Thai version of the ObsQoR-10 (ObsQoR-10-Thai) in patients who had undergone elective cesarean delivery. We hypothesized that the ObsQoR-10-Thai would have similar validity, reliability, responsiveness, and clinical feasibility to the original for assessing postoperative recovery after elective cesarean delivery.

## Methods

### Patient population

This prospective observational study was approved by the ethics committee of Ramathibodi Hospital, Mahidol University, Bangkok, Thailand (ID MURA2020/1939), and was registered on the Thai Clinical Trials Registry, identifier TCTR20210204001, registered on 04/02/2021 (Prospectively registration).

All participants provided written informed consent before their inclusion in the study. The study was conducted in accordance with the Declaration of Helsinki and followed the Strengthening the Reporting of Observational Studies in Epidemiology (STROBE) guidelines [16]. We included women aged > 18 years with American Society of Anesthesiologists (ASA) physical status class I–III who were scheduled to undergo cesarean delivery from February 2021 to April 2021. Patients were excluded if they were unable to read or listen, had a psychiatric disturbance that precluded complete cooperation, had a history of alcohol or drug dependence, had any severe preexisting medical condition that limited objective assessment after delivery, were undergoing emergency cesarean delivery, or were unwilling to participate in the study.

### Translation and cross-cultural adaptation of the ObsQoR-10

After receiving permission from the authors who created the original ObsQoR-10 to translate and modify, a three-step process to translate the original version into Thai was conducted based on recommendations [17] and previous validation studies [5, 7, 8]. First, two authors (S.D. and L.S.) translated the ObsQoR-10 into Thai. Second, the translated version was back translated into English-by-English linguistic academicians. Third, each question was rendered in its most comprehensible form by a committee of five people: two anesthesiologists, one obstetrician, one psychiatrist, and one health professional fluent in English. Each item of the back-translated questionnaire was assessed for its degree of agreement with the original questionnaire by using a three-point scoring system (0, 1, − 1), with 1 indicating absolute agreement and − 1 indicating absolute disagreement. Any items that scored more than 0.5 points were considered good agreement, whereas items that did not meet these criteria were reviewed. The final ObsQoR-10-Thai is available in Supplement 1.

After obtaining the ObsQoR-10-Thai, the pilot test was conducted on 20 randomly selected patients, who were not included in the study. The questionnaire did not need any further modifications after the pilot test. With this result, the ObsQoR-10-Thai was finalized.

### Study protocol

The day before surgery, patients were informed about the study and written consents for participation were obtained. Then the patients were asked to complete the ObsQoR-10-Thai, the activities of daily living (ADL) checklist, and a 100-mm visual analog scale of global health (VAS-GH) to evaluate their baseline status. The patients filled the three questionnaires again at 24 and 48-h postpartum.

The ADL checklist is a self-evaluation of independence that consists of six basic daily activities: bathing, dressing, toileting, transferring, continence, and feeding. The minimum and maximum ADL scores were 6 and 12, respectively, with higher scores indicating higher levels of independence in performing ADL. The VAS-GH is a global assessment of global health, where 0 and 100 indicate the worst and best health state, respectively. We also recorded the details of patient characteristics, including age, underlying disease, parity, education, type and duration of surgery, and length of hospital stay.

### Statistical analysis

#### Sample size estimation

The sample size was calculated based on the previous studies [11, 15], assuming a 10% dropout rate. Therefore, the required sample size was determined to be at least 110 participants.

### Data analysis

Data are presented as mean ± SD, median (interquartile range: IQR), number (%), or 95% CI. The Pearson and Spearman correlation analyses was used for normally and nonnormally distributed variables, respectively, with a correlation coefficient of 0.4–0.7 indicating a moderate correlation and 0.7–1.0 indicating a strong correlation). Reliability was assessed using Cronbach’s alpha (α) and intraclass correlation coefficients. Changes in the ObsQoR-10-Thai score among the three time points were compared using a multilevel mixed-effects linear regression and presented as mean difference and 95% confidence interval. All statistical analyses were performed using STATA version 17.0 (StataCorp. 2021. Stata Statistical Software: Release 17. College Station, TX: StataCorp LLC.). *P* < 0.05 was set as significant.

### Validation and comparison

The validity, reliability, and clinical feasibility of the ObsQoR-10-Thai were compared with the ADL questionnaire and VAS-GH.

Discriminant validity was measured by comparing the scores of the ObsQoR-10-Thai and the VAS-GH. Good and poor postoperative recovery were defined as VAS-GH of ≥ 70 and < 70 mm, respectively.

Convergent validity was measured by analyzing the correlation between the ObsQoR-10-Thai questionnaire with VAS-GH and the ADL questionnaire.

Construct validity indicated the associations between the ObsQoR-10-Thai and ASA physical status, operative time, blood loss, perioperative complications, and length of hospital stay.

Reliability testing: Reliability was assessed using the internal consistency (Cronbach’s alpha) of the ObsQoR-10-Thai at the three time points. The correlation between two segments of the ObsQoR-10-Thai was also analyzed. Test–retest reliability was evaluated by repeating the ObsQoR-10-Thai in a subset of 30 patients within 30–60 min. The correlation between measurements was then assessed. Floor and ceiling effects of the ObsQoR-10-Thai were assessed by calculating the proportion respondents that achieved the highest (100) or lowest (0) possible scores (acceptable: < 15%).

Acceptability and feasibility were measured by using the time taken for patients to complete the questionnaire (measured and recorded by the investigator), recruitment, and successful completion rate.

## Results

### Demographic data

A total of 110 patients were recruited, and there were no refusals. All patients completed the ObsQoR-10-Thai, ADL, and VAS-GH questionnaires. There were no incomplete or missing data, and no patients were excluded.

The mean patient age was 33.6 ± 4.4 years, and 108 patients (98%) were categorized as ASA PS class 2. Fifty-three patients (48%) had a history of previous cesarean delivery. One hundred and five patients (95%) underwent cesarean section under spinal anesthesia. The median length of hospital stay was 4 days (IQR 4, 5) days. The baseline characteristics are presented in Table 1.

**Table 1:** Patients’ clinicodemographic characteristics

Variables	*n* = 110
Age, year, mean (SD)	33.6 (4.4)
Gestational age, weeks, mean (SD)	38.1 (0.8)
Body weight, kg, mean (SD)	69.9 (12.0)
Maternal education, n (%)	
∙ Less than high school	2 (1.8)
∙ High school	6 (5.5)
∙ Bachelor’s degree	66 (60.0)
∙ Master’s degree	28 (25.5)
∙ Others	8 (7.3)
Anesthetic technique, n (%)	
∙ General anesthesia	2 (1.8)
∙ Spinal anesthesia	105 (95.5)
∙ Epidural anesthesia	1 (0.9)
∙ Others	2 (1.8)
ASA PS class, n (%)	
∙ II	108 (98.2)
∙ III	2 (1.8)
Previous cesarean delivery, n (%)	53 (48.6)
Medical conditions, n (%)	
∙ Hypertensive disorder, n (%)	3 (2.7)
∙ Gestational diabetes, n (%)	20 (18.2)
∙ Obesity, n (%)	8 (7.3)
∙ Dyslipid, n (%)	2 (1.8)
∙ Others, n (%)	14 (12.7)
Indication for cesarean delivery	
∙ Previous cesarean delivery, n (%)	53 (48.6)
∙ Breech presentation, n (%)	9 (8.2)
∙ Abnormal placenta, n (%)	4 (3.6)
∙ Multiple pregnancy, n (%)	3 (2.7)
∙ Others, n (%)	41 (37.3)
Type of operation	
∙ Cesarean delivery	81 (73.6)
∙ Cesarean delivery with tubal ligation	28 (22.5)
∙ Cesarean delivery with hysterectomy	1 (0.9)
Operative time, mean (SD)	80.8 (30.8)
Estimated blood loss, median (IQR)	400.0 (300.0, 500.0)
Perioperative complication, n (%)	8 (7.3)
Post-partum hemorrhage (estimated blood loss ≥ 1,000 ml)	
∙ Post-operative ICU admission, n (%)	1 (0.9)
∙ Length of hospital stay, median (IQR)	4 (4–5)

*ASA PS* American society of anesthesiologists, *ICU* Intensive care unit, *GA*, General anesthesia, *IQR* Interquartile range, *Kg* Kilogram, *ML* Milliliter, *SD* Standard deviation

### Recovery score before and after cesarean delivery

**Fig. 1:**
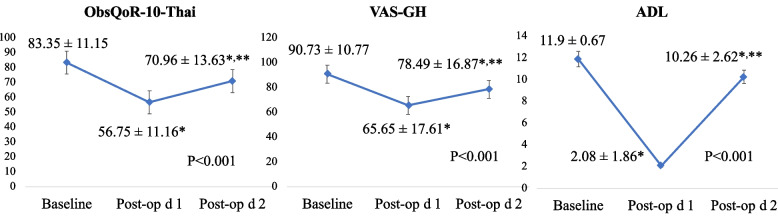
Changes in the ObsQoR-10-Thai, ADL, and global VAS-R scores at baseline, 24 and 48-h postpartum

The mean ObsQoR-10-Thai, ADL, and VAS-GH scores are summarized in Fig. 1. All scores were lower at both postoperative times than the baseline values, with the lowest scores at 24-h postpartum.

**Fig. 2:**
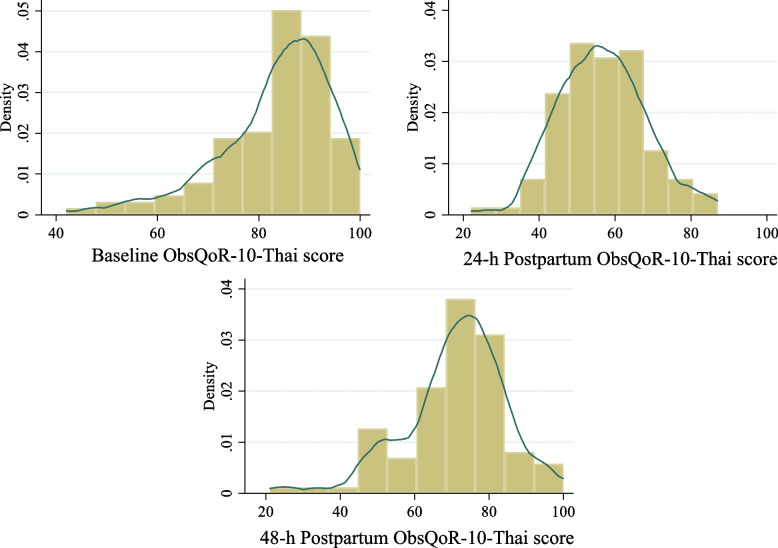
Histogram of the ObsQoR-10-Thai score at baseline, 24 and 48-h postpartum

The 24-h median ObsQoR-10-Thai score was 57 (IQR 48.5, 63.5), which was the lowest of the three time points. The items performing best to worst over the 48-h postpartum study period are listed in Table 2. The skewness and kurtosis values of the baseline ObsQoR-10-Thai scores were − 0.3 and 2.4, respectively, and those at 24 and 48-h postpartum were 0.19 and 3.26 and − 0.79 and 4.53, respectively (Fig. 2).

**Table 2:** Individual items score of ObsQoR-10-Thai at baseline, 24-h and 48-h postpartum

**Score**	**Baseline** **Median (IQR)**	**24-h** **median (IQR)**	**48-h** **median (IQR)**
**1 (highest ranked)**	Shivering 10 (10,10)	Shivering 10 (10, 10)	Shivering 10 (10, 10)
**2**	Nausea/vomiting 10 (10, 10)	Nausea/vomiting 10 (8, 10)	Nausea/vomiting 10 (10, 10)
**3**	Dizziness 10 (10, 10)	Dizziness 9 (8, 10)	Dizziness 10 (10, 10)
**4**	Pain 10 (9, 10)	Control 8 (5, 9)	Control 8 (7, 10)
**5**	Hygiene 9.5 (8, 10)	Pain 6 (5, 8)	Hygiene 8 (5, 9)
**6**	Control 9 (7, 10)	Comfortable 5 (4, 6)	Pain 7 (5, 7)
**7**	Move independently 8 (6, 9)	Move independently 4 (3, 5)	Move independently 6 (5, 7)
**8**	Comfortable 8 (6, 9)	Hygiene 3 (0, 5)	Comfortable 5 (5, 7)
**9**	Hold baby 7 (5, 8)	Hold baby 3 (0, 4)	Hold baby 5 (3, 7)
**10 (lowest ranked)**	Feed and nurse baby 7 (5, 8)	Feed and nurse baby 2 (0, 5)	Feed and nurse baby 5 (3, 7)

*IQR* Interquartile range

### Reliability and validity

Discrimination validity was assessed by comparing the ObsQoR-10-Thai score in women who had a good (VAS-GH ≥ 70) or poor (VAS-GH < 70) recovery status. The mean ObsQoR-10-Thai scores at 24 h were 75.58 (± 13.81) for good health state vs. 52.56 (± 10.61) for poor health state (*P* < 0.001).

Convergent validity was assessed using the correlation between the ObsQoR-10-Thai and the VAS-GH. ObsQoR-10-Thai correlated moderately with VAS-GH at predelivery (*r* = 0.60, *P* < 0.001), 24-h (*r* = 0.45, *P* < 0.001) and 48-h postpartum (*r* = 0.59, *P* < 0.001). However, the ADL was correlated with ObsQoR-10 only at predelivery (0.45, *P* < 0.001) and 48-h postpartum (0.42, *P* < 0.001) (Table 3).

**Table 3:** Correlation coefficients of ObsQoR-10-Thai with ADL and VAS-GH

**Correlation coefficient rho**		
Thai ObsQoR-10	**VAS-GH**	**ADL**
**Baseline**	0.60 (*P* < 0.001)	0.45 (*P* < 0.001)
**24-h postpartum**	0.45 (*P* < 0.001)	0.07 (*P* 0.449)
**48-h postpartum**	0.59 (*P* < 0.001)	0.42 (*P* < 0.001)

*ADL* Activities of daily living, *ObsQoR-10-Thai* Obstetric quality of recovery 10, *VAS-GH* Visual analog scale of global health (VAS-GH)

The ObsQoR-10-Thai did not correlate with ASA physical status (*r* = 0.14, *P* = 0.14), operative time (*r* = 0.12, *P* = 0.19), blood loss (*r* = 0.13, *P* = 0.17), perioperative complications (*r* = 0.01, *P* = 0.94), and length of hospital stay (*r* = – 0.07, *P* = 0.92).

The interdimensional correlation matrices of the postdelivery ObsQoR-10-Thai are presented in Table 4. Before delivery and 24 and 48-h postpartum, the interitem Cronbach α values were 0.80, 0.67, and 0.87, respectively, and the split-half correlations were 0.88, 0.78, and 0.92, respectively. The test–retest reliability of the ObsQoR-10-Thai was 0.99 (95% CI: 0.98–0.99). No participant had a score of 0 or 100, which was well within the acceptable limit of < 15%.

**Table 4:** Interdimensional correlations for postoperative ObsQoR-10-Thai scores by items

Thai ObQoR-10	VAS-GH	Total score	1	2	3	4	5	6	7	8	9	10
**1) Nausea or vomiting**	0.41 (0.24, 0.55) *P* < 0.001)	0.60	-									
**2) Dizziness**	0.10 (-0.01, 0.28) *P* = 0.314	0.36	0.20	-								
**3) Shivering**	0.35 (0.17, 0.50) *P* > 0.001	0.62	0.47	0.43	-							
**4) Feeling in control**	0.16 (-0.03, 0.34) *P* = 0.088	0.41	0.19	0.66	0.49	-						
**5) Comfortable**	0.55 (0.41, 0.67) *P* < 0.001	0.75	0.44	0.25	0.41	0.26	-					
**6) mobilize independently**	0.53 (0.38, 0.65) *P* < 0.001	0.86	0.51	0.23	0.41	0.29	0.78	-				
**7) Hold baby without assistance**	0.40 (0.24, 0.55) *P* < 0.001	0.83	0.33	0.13	0.29	0.16	0.56	0.69	-			
**8) Feed/ nurse baby without assistance**	0.34 (0.17, 0.50) *P* < 0.001	0.81	0.30	0.15	0.33	0.19	0.48	0.67	0.93	-		
**9) I can look after my personal hygiene/ toilet**	0.49 (0.34, 0.62) *P* < 0.001	0.73	0.33	0.16	0.37	0.25	0.43	0.55	0.53	0.55	-	
**10) Pain**	0.52 (0.37, 0.64) *P* < 0.001	0.66	0.28	0.17	0.42	0.20	0.46	0.48	0.47	0.43	0.46	-

*ObsQoR-10-Thai* Obstetric quality of recovery 10, *VAS-GH* Visual analog scale of global health

The clinical acceptability and feasibility of the ObsQoR-10-Thai were excellent, with a 100% completion rate. The median time to complete the questionnaire was 2 (1–6) min.

## Discussion

Our study demonstrates that the ObsQoR-10-Thai has good validity and is consistent with the original ObsQoR-10. The ObsQoR-10-Thai score did not correlate with ASA physical status, perioperative complications, operative time, blood loss, or length of hospital stay. However, the internal consistency measured by Cronbach α, and split-half reliability was excellent, with a high degree of clinical feasibility and responsiveness. Our findings indicate that the ObsQoR-10-Thai provides a valid, reliable, and responsive global assessment of health state after elective cesarean delivery.

The ObsQoR-11 was first developed and evaluated for elective cesarean delivery at a single UK university hospital, with a moderate correlation between ObsQoR-11 and global health status (*r* = 0.53, *P* < 0.001) [5]. The ObsQoR-10 was demonstrated to have moderate correlation with global VAS (*r* = 0.57, 0.51, and 0.45 in Brazilian [13], US (8), and Indian (11) populations, respectively) and EuroQoL (*r* = –0.59 and –0.51 in Brazilian (13) and US (8) populations, respectively). The ObsQoR-10-Thai also had a moderate correlation with the global VAS score (0.45–0.6), suggesting that the ObsQoR-10-Thai is useful for measuring the quality of recovery after elective cesarean delivery in our setting. Compared with ADL, the ObsQoR-10-Thai had higher correlation coefficients at baseline and 24 and 48 h postpartum, which emphasized the ability to measure the quality of recovery better than the ADL score. The ObsQoR-10-Thai also appears to discriminate well between “good” or “poor” recovery, as defined by the global VAS scores of ≥ 70 and < 70, respectively. However, the reliability of the ObsQoR-10-Thai in our setting was at a moderate level, warranting further interdimensional analysis.

In our cohort, the median 24-h ObsQoR-10-Thai was 57, which was lower than that reported in the US (median: 77) [8] and Brazilian populations (median: 84) [13]. In both the Thai and Indian cohorts of women undergoing elective cesarean delivery, dizziness and nausea/vomiting were among the three highest ranked ObsQoR recovery items. The lowest three ranked items in the Thai cohort were the ability to hold the baby, look after their personal hygiene, and mobilize and in the US cohort were pain, shivering, and dizziness [8]. The lowest ranked item in both the US and Brazilian studies was pain, with mean scores of 5.8 [8] and 6.6 [13], respectively. Consistently, our mean pain score outcome was 6. Since the ObsQoR-10 is a patient-reported outcome measurement (PROM), which is influenced by socioeconomic, linguistic, and cultural factors [18–20]. Moreover, recovery outcomes are also likely to be influenced by anesthesia drug regimens, hospital protocols, and surgical factors. Therefore, these factors may affect the rankings or scores among different counties.

Our data indicated that the ObsQoR-10-Thai did not correlate with maternal age, ASA physical status, operative time, estimated blood loss, perioperative complications, or length of hospital stay. This is consistent with previous studies [8, 10, 13]. This could be a limitation of the clinical validity of the ObsQoR-10-Thai in this setting. However, this may also be because our population was quite healthy, with a low incidence of complications or outcome variability. This may have made the study underpowered to detect the differences. For example, only eight women in our study had an estimated blood loss ≥ 1,000 mL, and only one required admission to the intensive care unit after delivery. Moreover, hospital protocols and surgical or anesthesia factors may influence the results. For example, the patients have to be admitted to the hospital preoperatively and discharged on postoperative day 3 as an institute standard practice. Therefore, the differences in clinical practice may influence the clinical validation of the ObsQoR-10.

Recovery after delivery is a complex process, which depends on patient, surgical, and anesthetic characteristics; moreover, it means different things to different stakeholders [21]. A patient-centric approach is useful to integrate the patient’s role of perioperative care into a cohesive pathway to healthcare system. Multidimensional measurement of the quality of care directly benefits patients and facilitates auditing and improving healthcare-related service. The ObsQoR-10 has been included in a core outcome set of enhanced recovery after cesarean delivery outcomes in future and quality improvement endeavors. Therefore, a translated version of this tool in Thai will facilitate the implementation of the new core outcome set in Thailand. [22] Moreover, our study is the first to validate the ObsQoR-10 in a Southeast Asian country; this region has significantly different demographic, socioeconomic, and cultural characteristics than those of Western countries. The ObsQoR-10-Thai may be helpful for routine clinical use as a postpartum recovery assessment tool in conjunction with medical record evaluation and focused clinical examinations.

The strengths of our study are consistent measurement of recovery scores (ObsQoR-10-Thai and ADL) over 48 h postpartum. However, this study has some limitations. First, our main population had low ASA physical status and a low incidence of adverse outcomes (e.g., postpartum hemorrhage). Therefore, ObsQoR-10-Thai failed to correlate with ASA physical status, estimate blood loss, operative time, perioperative complications, and length of hospital stay. Second, our study was performed in a single-center tertiary university hospital. Third, we included only patients undergoing elective cesarean delivery. Future studies should evaluate the ObsQoR-10-Thai in other hospital settings and delivery modes to determine its generalizability. Fourth, the timing of follow-up was not standardized and may have coincided with variable timings depending on when the analgesia was given before or after attempted mobilization. Finally, we did not assess the neonatal health state, although the ability to nurse or hold the baby is included in the ObsQoR-10-Thai.

## Conclusions

The ObsQoR-10-Thai was found to be a valid, reliable, and clinically feasible patient-reported outcome measurement, which can be administered to Thai-speaking women undergoing elective cesarean delivery. Despite some limitations in construct validity, the ObsQoR-10-Thai performed had good convergent and discriminant validity and was found to be feasible and reliable. Further studies should evaluate the ObsQoR-10-Thai in an emergency setting or vaginal delivery.

## Supplementary Information

**Additional file 1. **Obstetric Quality of Recovery-10 (ObsQoR-10) Questionnaire.

**Additional file 2: Supplementary** **2.** Description of the perioperative protocol for elective caesarean deliveries.

## Data Availability

The datasets during and/or analyzed during the current study available from the corresponding author on reasonable request.

## Abbreviations

ADL: The activities of daily living
ASA PS status: American society of anesthesiologists physical status
CI: Confident interval
H: Hour
IQR: Interquartile range
ObsQoR: Obstetric Quality of Recovery score
PACU: Post anesthetic care unit
PROM: Patient-reported outcome measurement
SD: Standard deviation
US: United state
UK: United Kingdom
VAS-R: Visual analog scale of recovery status
