# Supplementary material for: Validation of the Thai version of the obstetric quality of recovery score (obsqor-10-Thai) after elective cesarean delivery
Source: BMC Anesthesiol. 2023 Mar 7;23:72. doi: 10.1186/s12871-023-02010-6 (PMC9990285; doi:10.1186/s12871-023-02010-6)
Supplement: Supplementary file 1 — Additional file 1. Obstetric Quality of Recovery-10 (ObsQoR-10) Questionnaire. [file 12871_2023_2010_MOESM1_ESM.docx]

**Obstetric Quality of Recovery-10 (ObsQoR-10) Questionnaire**

**How have you been feeling in the last 24 hours?**

**(0-10, where 0 = very poor and 10 = excellent)**

|  | None Moderate Worst imaginable  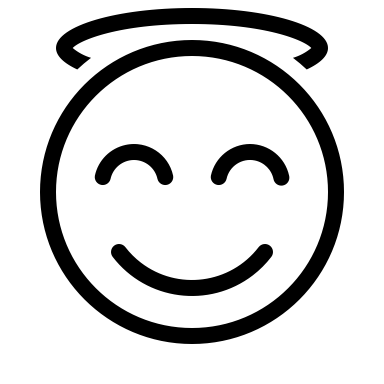 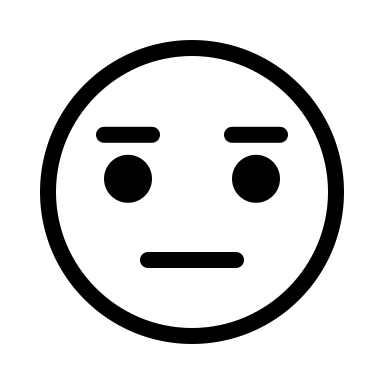 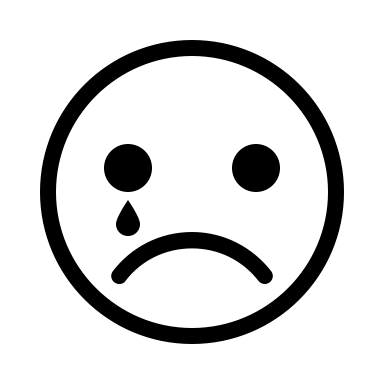 |
| --- | --- |
| 1. I have had pain | 10 9 8 7 6 5 4 3 2 1 0 |
| 1. I have had nausea or vomiting | 10 9 8 7 6 5 4 3 2 1 0 |
| 1. I have been feeling dizzy | 10 9 8 7 6 5 4 3 2 1 0 |
| 1. I have had shivering | 10 9 8 7 6 5 4 3 2 1 0 |
|  | **No/ Never Sometimes/ with help Yes/ Always**  **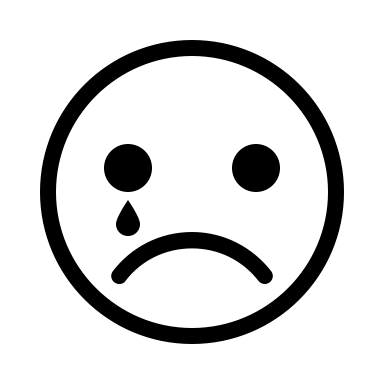 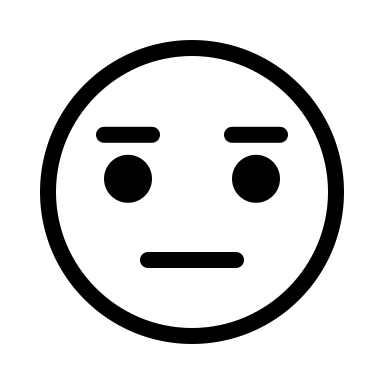 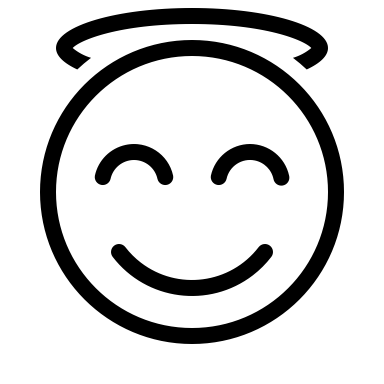** |
| 1. I have been comfortable | 0 1 2 3 4 5 6 7 8 9 10 |
| 1. I am able to mobilize independently | 0 1 2 3 4 5 6 7 8 9 10 |
| 1. I can hold baby without assistance | 0 1 2 3 4 5 6 7 8 9 10 |
| 1. I can feed/nurse my baby without assistance | 0 1 2 3 4 5 6 7 8 9 10 |
| 1. I can look after my personal hygiene/ toilet | 0 1 2 3 4 5 6 7 8 9 10 |

Global health score

Worse imaginable Health status Best imaginable health status

Please mark with an arrow or a cross on the scale below how you have been feeling in the last 24 hours
