# Supplementary material for: Validation of the Thai version of the obstetric quality of recovery score (obsqor-10-Thai) after elective cesarean delivery
Source: BMC Anesthesiol. 2023 Mar 7;23:72. doi: 10.1186/s12871-023-02010-6 (PMC9990285; doi:10.1186/s12871-023-02010-6)
Supplement: Supplementary file 2 — Additional file 2: Supplementary 2. Description of the perioperative protocol for elective caesarean deliveries. [file 12871_2023_2010_MOESM2_ESM.docx]

**Supplementary 2: Description of the perioperative protocol for elective caesarean deliveries**

Our institutional neuraxial anesthesia regimen includes intrathecal administration of 0.5% hyperbaric bupivacaine 2-2.5 ml and morphine 0.1-0.2 mg via a single shot spinal anesthesia for elective caesarean deliveries. All parturient receive 1-gram intravenous (IV) paracetamol and 40 mg IV parecoxib at the time of skin closure. Prophylactic antiemetic (intravenous ondansetron 4 mg) is given to all parturient at the end of the surgery prior to the postoperative ward. For postoperative analgesia, woman received regular paracetamol 500 mg four times daily and ibuprofen 400 mg every 8 hours unless contraindicated, and for breakthrough pain as required tramadol 50 mg IV six-hourly on the day of surgery and morphine 3 mg four-hourly from day one postoperatively. Intravenous ondansetron 4-8 mg six-hourly as required were also prescribed unless contraindicated.
